# Toward the Role of L2 Enjoyment in EFL Students' Academic Motivation and Engagement

**DOI:** 10.3389/fpsyg.2021.822588

**Published:** 2022-01-26

**Authors:** Shanshan Liu

**Affiliations:** Foreign Languages Department, Liaocheng University Dongchang College, Liaocheng, China

**Keywords:** academic motivation, academic engagement, L2 enjoyment, EFL students, emotional factor

## Abstract

Since students' academic success is tied to their academic motivation and engagement, determining the predictors of these two variables seems critical. So, several inquiries have inspected the role of students' emotional and personal variables in their academic motivation and engagement. Nonetheless, the function of L2 enjoyment as an important emotional factor has remained elusive. Moreover, no inquiry has reviewed this issue neither systematically nor theoretically. To fill these lacunas, this review study aims to explain definitions, dimensions, and theoretical frameworks of L2 enjoyment, student academic motivation, and student academic engagement. This study also intends to outline the positive consequences of L2 enjoyment for students' academic engagement and motivation. Relying on the theoretical and empirical evidence, the positive role of L2 enjoyment in raising EFL students' engagement and motivation was firmly affirmed. Finally, the implications of the findings are discussed.

## Introduction

Academic motivation and engagement are inseparable aspects of students' success in educational settings (Roksa and Whitley, 2017; Kahu and Nelson, 2018). That is, unmotivated and disengaged students will find nothing but academic failure (Peng, 2021). Because of this, improving students' motivation and engagement has always been the primary concern of all distinguished teachers. However, many university lecturers, instructors, and teachers, including EFL teachers, have no idea how to raise their pupils' motivation and academic engagement (Ford, 2012). Students' academic motivation in a general term pertains to “individual students' primary impetus for initiating learning as well as the reason for continuing the prolonged and tedious process of learning” (Ushioda, 2008, p. 19). Language learners' motivation, in particular, refers to students' attempts and desire to achieve the goals of acquiring the language (Guilloteaux and Dörnyei, 2008). In a similar definition, Hsu (2010) described this construct as the extent to which a language learner puts effort into acquiring a language due to his/her personal desire to do so and a sense of happiness and satisfaction experienced throughout the learning process. Besides, academic engagement as another influential factor in students' learning success pertains to “the quality and quantity of students' participation or connection with the educational endeavor and hence with activities, values, individuals, aims, and place that comprise it” (Skinner et al., 2009, p. 496). According to Baralt et al. (2016), the construct of academic engagement in the language education domain refers to the amount of students' participation in language-related activities and their continual endeavors in mastering a foreign/second language. Taken together, academic motivation and academic engagement are two related concepts (Ghelichli et al., 2020) that are intertwined with students' progress, academic growth, and increased achievement (Zhou et al., 2021).

To illustrate the value of students' academic motivation, Gilakjani et al. (2012) stated that academic motivation as an important personal trait plays a facilitative role in promoting students' academic performance. Cheng and Cheng (2012) also submitted that students with high levels of academic motivation typically have positive attitudes toward learning, which empowers them to proceed with the learning process successfully. In a similar vein, Feng et al. (2013) mentioned that students' learning outcomes can be remarkably affected by their academic motivation. To them, motivated students typically gain more desirable outcomes as a result of the extra effort they devote to classroom tasks and activities. In this regard, Yin and Wang (2016) also explained the value of academic motivation by highlighting its positive consequences for students' classroom engagement. They posited that academic motivation as a driving force stimulates students to take an active role in classroom contexts.

In an effort to illuminate the importance of students' academic engagement, Liem and Chong (2017) stated that students' educational attainment highly depends on the extent to which they are committed to and take part in classroom activities. In a similar attempt, Hiver et al. (2021) also referred to the value of academic engagement by revealing its capability to predict a range of positive educational outcomes such as academic growth, advancement, and success. Additionally, Carver et al. (2021) suggested that students' active involvement in educational contexts will gradually improve their learning outcomes. Based on what has been noted on the significance of students' academic engagement and motivation, delving into the antecedents of these interrelated constructs appears to be critical.

Against this backdrop, a multitude of empirical and theoretical inquiries have been dedicated to students' academic motivation and engagement and their determinants. Several studies have inspected the effects of teachers' personal (e.g., self-efficacy, autonomy, personality, etc.) and interpersonal traits (e.g., praise, caring, stroke, credibility, immediacy, confirmation, etc.) on students' level of motivation (Furlich, 2016; Wijaya, 2017; Sabet et al., 2018; Khalilzadeh and Khodi, 2021; Liu, 2021; Peng, 2021; Xie and Derakhshan, 2021, to cite a few) and engagement (Imlawi et al., 2015; Papa, 2015; Derakhshan, 2021; Gao, 2021; Lu and Mustafa, 2021; Wang and Ye, 2021; Zheng, 2021; Zhou, 2021, to cite a few). Many studies have also delved into the impact of students' personal (e.g., self-efficacy, character, etc.) and emotional factors (e.g., satisfaction, happiness, joy, etc.) on these constructs (Ariani, 2015; Qureshi et al., 2016; Zhang et al., 2020b; Angelovska et al., 2021; Chen et al., 2021; Khajavy, 2021, to cite a few). Yet, L2 enjoyment as a prime instance of students' emotional factor has received scant attention (Dewaele and Proietti Ergn, 2020; D'Orazzi, 2020; Zhang et al., 2020a; Wang et al., 2021).

The concept of enjoyment in a broad term refers to the degree to which an experience, event, or action is perceived as offering joy, happiness, and pleasure (Brantmeier, 2005). Further, Csikszentmihalyi (2014) conceptualized this construct by distinguishing it from happiness and pleasure. To him, enjoyment is generally “a good feeling coming from breaking through homeostatic limits and stretching beyond oneself to accomplish something new or even unexpected, especially in face of some difficult tasks” (as cited in Li et al., 2018, p. 5). In a similar vein, Dewaele and MacIntyre (2016) defined L2 enjoyment as “a complex emotion, capturing interacting dimensions of the challenge and perceived ability that reflects the human drive for success in the face of difficult tasks” (p. 216). According to Dewaele et al. (2018), L2 enjoyment arises as students meet their educational needs and even go beyond them to gain something new throughout the process of language learning. As Dewaele and MacIntyre (2019) noted, L2 Enjoyment as a positive activating disposition drives students into action and strengthens their educational motives. Lee (2020) also proposed that L2 enjoyment can improve language learners' tendency to take part in classroom activities.

In spite of the importance of L2 enjoyment in raising students' academic engagement and motivation (Dewaele and MacIntyre, 2019; Lee, 2020; Wang and Guan, 2020), not much scholarly attention has been paid to the association of these variables (Dewaele and Proietti Ergn, 2020; D'Orazzi, 2020; Zhang et al., 2020b). Furthermore, no comprehensive review has been done on this topic. This review study is an attempt to address these gaps by offering a through description of these constructs (i.e., L2 enjoyment, academic motivation, and academic engagement), their theoretical underpinnings, and their associations.

### Student Academic Motivation

Motivation in a general sense is the underlying reason for humans' behaviors (Covington and Müeller, 2001). That is, motivation provides people with a purpose, direction, and orientation to pursue (Broussard and Garrison, 2004). Dörnyei and Ushioda (2009) conceptualized student academic motivation as “individual students' motive to make certain academic decisions, participate in classroom activities, and persist in pursuing the demanding process of learning” (p. 2). To enumerate different types of student academic motivation, Ryan and Deci (2000) stated that academic motivation as an incentive prompting students to pursue their academic goals is of two types, namely “*intrinsic/internal motivation*” and “*extrinsic/external motivation*”. Students' intrinsic motivation pertains to the sense of satisfaction and enjoyment they gain while performing academic tasks/activities (Osterloh and Frey, 2000). Students' extrinsic motivation also refers to the benefits and rewards they receive as a result of their actions (Pintrich and Schunk, 2002).

### Student Academic Engagement

Student engagement generally includes the amount of time and energy students freely and enthusiastically allocate to their learning (Hu and Kuh, 2002; Kuh, 2003). Lawson and Lawson (2013) defined this concept as students' physical and psychological investments toward acquiring, comprehending, and mastering the knowledge, content, and information that instructors strive to convey. Student engagement, as Skinner and Pitzer (2012) mentioned, deals with “how actively involved a student is in a learning task and the extent to which that physical and mental activity is goal-directed and purpose-driven” (p. 22).

There has been no agreement among scholars on how to name this concept (Alrashidi et al., 2016). That is, different scholars named this construct in various ways. Many academics, however, referred to this concept as “*student academic engagement”* (Greenwood et al., 2002; Furrer and Skinner, 2003; Ross et al., 2008; to cite a few). The rest named it as “*educational engagement”* (MacDonald and Marsh, 2004; London et al., 2007), “*study engagement”* (Ouweneel et al., 2011; Salmela-Aro and Read, 2017), and “*school engagement”* (Fredricks et al., 2005; Dotterer and Lowe, 2011).

Likewise, scholars have reached no consensus regarding the categorization of student engagement. It means that they classified the components of this construct in different ways. Finn (1989), for instance, divided the underlying factors of student engagement into two major categories, namely “*behavioral engagement*” and “*emotional engagement*”. In contrast, Jimerson et al. (2003) grouped the components of this construct into three dimensions of “*affective engagement*”, “*behavioral engagement*”, and “*cognitive engagement*” (Table 1).

**Table 1:** Different categorizations of student engagement.

**Scholars**	**Underlying components**
Finn (1989)	Behavioral engagement: the quality and quantity of students' in-class participation Emotional engagement: students' sense of connectedness
Jimerson et al. (2003)	Behavioral engagement: students' constant attention, perseverance, and endeavor Cognitive engagement: student's use of learning strategies Affective engagement: students' positive viewpoints and/or attitudes toward their peers, instructors, and learning environment

Regardless of the type and the number of its underlying components, student academic engagement is a multi-faceted construct that represents students' academic inclination (Upadyaya and Salmela-Aro, 2013).

### L2 Enjoyment

Enjoyment as an emotional experience is associated with a range of positive feelings, including pleasure, joy, and contentment (Goetz et al., 2006; Guo, 2021). Enjoyment in language classes, which is known as L2 enjoyment, is conceptualized as “a complex emotion, capturing interacting dimensions of challenge and perceived ability” (Dewaele and MacIntyre, 2016, p. 217). As Dewaele and MacIntyre (2016) noted, L2 enjoyment has two major dimensions, namely “*social dimension*” and “*private dimension*”. The social dimension of L2 enjoyment is tied to a desirable learning atmosphere as well as supportive and encouraging instructors, contributing to students' positive emotions. The private dimension is also related to the cognition that comes with enjoyment (Dewaele and Dewaele, 2017). The private and social aspects of L2 enjoyment are intertwined and collaborate to generate a cohesive feeling (Dewaele and MacIntyre, 2014).

### The Role of L2 Enjoyment in EFL Students' Academic Motivation and Engagement

The role of L2 enjoyment in raising EFL students' academic engagement and motivation can be fully explained through “*Broaden-and-Build Theory* (BBT)” (Fredrickson, 2003). In BBT, Fredrickson (2003) submitted that positive feelings such as L2 enjoyment “broaden students' momentary thought-action repertoires and build their enduring personal resources” (p. 219). Accordingly, students' intrinsic motivation and academic engagement as instances of their personal resources can be strengthened as a result of positive emotions, including L2 enjoyment. In light of this theory, MacIntyre and Gregersen (2012) also stated that positive emotions such as L2 enjoyment prompt language learners to become engrossed in the learning process. Additionally, building upon “*Control-value Theory* (CVT)” (Pekrun et al., 2002), Dewaele and Li (2020) suggested that different L2 learning outcomes such as L2 motivation and academic engagement can be positively influenced by positive achievement emotions, including L2 enjoyment. In this regard, Kahu et al. (2015) also illustrated that in the presence of positive academic emotions such as L2 enjoyment, language learners tend to invest more time and effort toward learning a new language.

## Empirical Studies

As previously mentioned, despite the importance of L2 enjoyment in promoting EFL students' motivation and engagement, only a few scholars have inspected the associations of these constructs (Dewaele and Proietti Ergn, 2020; D'Orazzi, 2020; Zhang et al., 2020a). Dewaele and Proietti Ergn (2020), for instance, scrutinized the consequences of L2 enjoyment for EFL students' academic motivation. To this aim, 110 Turkish students were asked to fill out two close-ended scales designed to measure L2 enjoyment and students' level of motivation. The analysis of participants' answers revealed a positive connection between sense of enjoyment and academic motivation. By the same token, D'Orazzi (2020) also inspected the role of L2 enjoyment in increasing university students' motivation. In doing so, two valid measures of L2 enjoyment and academic motivation were given to 728 university students. The analysis of the gathered data indicated that L2 enjoyment can dramatically contribute to students' increased motivation. In a similar vein, Zhang et al. (2020b) studied L2 enjoyment in relation to Chinese EFL students' motivation. To this end, two pre-developed questionnaires were administered to 335 university students. Participants' responses to the questionnaires were thoroughly analyzed through IBM SPSS software. The results demonstrated a strong and favorable association between L2 enjoyment and student motivation.

## Conclusion and Pedagogical Implications

So far, definitions, dimensions, and theoretical frameworks of L2 enjoyment, student academic motivation, and student academic engagement were presented. Based on the “broaden-and-build theory” and “control-value theory”, the close bond between L2 enjoyment, student academic motivation, and student academic engagement was also illustrated. A short summary of the previous related articles was also provided. Drawing on the aforementioned theories and the results of previous studies, one can logically infer that L2 enjoyment as an emotional experience can greatly affect EFL students' motivation and engagement in a positive way. This result may be illuminating for EFL teachers, notably those who do not know how to improve their pupils' motivation and engagement. Given the centrality of positive emotions such as L2 enjoyment in EFL students' enhanced engagement and motivation (MacIntyre and Gregersen, 2012; Dewaele and Li, 2020), teachers are required to provide an appropriate learning environment wherein pupils can enjoy acquiring a second language. The review's findings seem to be informative for teacher educators as well. They are expected to instruct both pre-service and in-service instructors on how to instill a sense of enjoyment in their pupils.

## Data Availability Statement

The original contributions presented in the study are included in the article/supplementary material, further inquiries can be directed to the corresponding author/s.

## Ethics Statement

The studies involving human participants were reviewed and approved by Liaocheng University Dongchang College Research Ethics Committe. The patients/participants provided their written informed consent to participate in this study.

## Author Contributions

The author confirms being the sole contributor of this work and has approved it for publication.

## Funding

This manuscript was supported by Industry-University Collaborated Education Project: Business English Professional Teacher Training Under the Industry-University Cooperation Model (202002202012).

## Conflict of Interest

The author declares that the research was conducted in the absence of any commercial or financial relationships that could be construed as a potential conflict of interest.

## Publisher's Note

All claims expressed in this article are solely those of the authors and do not necessarily represent those of their affiliated organizations, or those of the publisher, the editors and the reviewers. Any product that may be evaluated in this article, or claim that may be made by its manufacturer, is not guaranteed or endorsed by the publisher.
